# Tolerability and safety of weekly primaquine against relapse of *Plasmodium vivax* in Cambodians with glucose-6-phosphate dehydrogenase deficiency

**DOI:** 10.1186/s12916-015-0441-1

**Published:** 2015-08-25

**Authors:** Sim Kheng, Sinoun Muth, Walter R. J. Taylor, Narann Tops, Khem Kosal, Khon Sothea, Phum Souy, Saorin Kim, Chuor Meng Char, Chan Vanna, Po Ly, Pascal Ringwald, Virak Khieu, Alexandra Kerleguer, Pety Tor, John K. Baird, Steven Bjorge, Didier Menard, Eva Christophel

**Affiliations:** National Center for Parasitology, Entomology and Malaria Control, Phnom Penh, Cambodia; Service de Médecine Tropicale et Humanitaire, Hôpitaux Universitaires de Genève, Geneva, Switzerland; Mahidol Oxford Tropical Medicine Research Unit, 420/60 Rajvithi Road, Bangkok, 10400 Thailand; WHO Cambodia Country Office, Pasteur Street, Phnom Penh, Cambodia; Pailin Referral Hospital, Pailin, Cambodia; Anlong Veng Referral Hospital, Anlong Venh, Oddar Meanchey Cambodia; Institut Pasteur in Cambodia, Phnom Penh, Cambodia; Pramoy Health Centre, Veal Veng, Pursat Cambodia; WHO Headquarters, Geneva, Switzerland; Eijkman Oxford Clinical Research Unit, Jakarta, Indonesia; Centre for Tropical Medicine, Nuffield Department of Medicine, University of Oxford, Oxford, UK; WHO Western Pacific Regional Office, Manila, Philippines

## Abstract

**Background:**

Primaquine is used to prevent *Plasmodium vivax* relapse; however, it is not implemented in many malaria-endemic countries, including Cambodia, for fear of precipitating primaquine-induced acute haemolytic anaemia in patients with glucose-6-phosphate dehydrogenase deficiency (G6PDd). Reluctance to use primaquine is reinforced by a lack of quality safety data. This study was conducted to assess the tolerability of a primaquine regimen in Cambodian severely deficient G6PD variants to ascertain whether a weekly primaquine could be given without testing for G6PDd.

**Methods:**

From January 2013 to January 2014, Cambodians with acute vivax malaria were treated with dihydroartemisinin/piperaquine on days (D) 0, 1 and 2 with weekly doses of primaquine 0.75 mg/kg for 8 weeks (starting on D0, last dose on D49), and followed until D56. Participants’ G6PD status was confirmed by G6PD genotype and measured G6PD activity. The primary outcome was treatment completion without primaquine toxicity defined as any one of: (1) severe anaemia (haemoglobin [Hb] <7 g/dL), (2) a >25 % fractional fall in Hb from D0, (3) the need for a blood transfusion, (4) haemoglobinuria, (5) acute kidney injury (an increase in baseline serum creatinine >50 %) or (6) methaemoglobinaemia >20 %.

**Results:**

We enrolled 75 patients with a median age of 24 years (range 5–63); 63 patients (84 %) were male. Eighteen patients were G6PDd (17/18 had the Viangchan variant) and had D0 G6PD activity ranging from 0.1 to 1.5 U/g Hb (median 0.85 U/g Hb). In the 57 patients with normal G6PD (G6PDn), D0 G6PD activity ranged from 6.9 to 18.5 U/g Hb (median 12 U/g Hb). Median D0 Hb concentrations were similar (*P* = 0.46) between G6PDd (13 g/dL, range 9.6–16) and G6PDn (13.5 g/dL, range 9–16.3) and reached a nadir on D2 in both groups: 10.8 g/dL (8.2–15.3) versus 12.4 g/dL (8.8–15.2) (*P* = 0.006), respectively. By D7, five G6PDd patients (27.7 %) had a >25 % fall in Hb, compared to 0 G6PDn patients (*P* = 0.00049). One of these G6PDd patients required a blood transfusion (D0–D5 Hb, 10.0–7.2 g/dL). No patients developed severe anaemia, haemoglobinuria, a methaemoglobin concentration >4.9 %, or acute kidney injury.

**Conclusions:**

Vivax-infected G6PDd Cambodian patients demonstrated significant, mostly transient, falls in Hb and one received a blood transfusion. Weekly primaquine in G6PDd patients mandates medical supervision and pre-treatment screening for G6PD status. The feasibility of implementing a package of G6PDd testing and supervised primaquine should be explored.

**Trial registration:**

The trial was registered on 3/1/2013 and the registration number is ACTRN12613000003774.

**Electronic supplementary material:**

The online version of this article (doi:10.1186/s12916-015-0441-1) contains supplementary material, which is available to authorized users.

## Background

The malaria parasite *Plasmodium vivax* causes an acute symptomatic blood-stage infection and characteristically has a liver stage of dormant parasites, called hypnozoites. Weeks to months later, these hypnozoites awaken to cause renewed blood infections, called relapses. Although considered a benign infection, acute vivax can result in severe disease and death in ~2 % of hospitalised patients [1, 2]. Treatment is further challenged by the increasing prevalence of vivax resistance to the commonly used chloroquine [1, 3].

Treatment to eliminate the hypnozoites is also challenging for several reasons. Most *P. vivax* strains in South-East Asia (e.g. Chesson strain from New Guinea) relapse early and frequently, typically within 3 weeks, and cause a median of 5–6 relapses/person-year [4]. Primaquine is the only licensed drug for eliminating the hypnozoites and high doses (0.5 mg/kg/day for 14 days) are needed for strains from South-East Asia [5–7]. Mutations in the gene encoding for the cytochrome P450 2D6 enzyme may result in fewer active oxidative metabolites and compromise the anti-relapse efficacy of primaquine [8]. In patients with the X-linked erythrocyte enzyme disorder glucose-6-phosphate dehydrogenase deficiency (G6PDd), primaquine causes dose-dependent acute haemolytic anaemia (AHA) that is greater in the more severe deficient G6PD variants; AHA can be potentially life threatening but primaquine-related deaths are very rare [9–15]. This toxicity is a significant public health concern because G6PDd affects approximately 400 million people who live mostly in malaria-endemic countries where the median G6PDd allele prevalence is 8 % [16]. Testing for G6PDd is not performed in the majority of malaria-endemic countries and this effectively blocks the use of primaquine.

In a pioneering report in 1960, Alving and colleagues experimentally challenged African Americans with G6PDd A^−^ with the Chesson strain of *P. vivax* and successfully treated them against relapse using a single weekly dose of 0.75 mg/kg (45 mg) of primaquine for eight weeks [17]. This regimen was well tolerated and produced only minor fractional drops in haemoglobin (Hb) [7 % versus day (D)0] compared to the steeper drops (35–50 % versus D0) in the same patients given daily primaquine [0.5 mg/kg (30 mg) × 14 days]. This regimen came to be viewed as not only a safe anti-relapse therapy for G6PDd patients, but also as a therapeutic option where G6PD status was unknown. However, in 1960, few understood the great genotypic and phenotypic variation in G6PDd and their differing susceptibilities to primaquine-induced AHA and, in 1981, Clyde pointed out the dangers of indiscriminate primaquine use in countries with severe G6PDd variants [18].

Cambodia has a G6PDd allele prevalence of 14 % and the Viangchan variant accounts for 90 % of all variants. The median G6PD enzyme activity is 0.8 U/gHb, ~7 % of the median population value of 12 U/gHb [19, 20], making Viangchan a mostly class II G6PDd variant (i.e. 1 to <10 % of the population median [21]). When tested in fit and healthy G6PDd Cambodian airmen for 14 days, 15 mg of daily primaquine (half the Chesson strain dose) was tolerated despite causing a mean fall in haematocrit of 9 % (~3 g/dL Hb) from 43 % to 34 % on D7, representing a mean fractional drop of 21 % compared to day 0; two men had D7 haematocrits of 26 and 28 % [22]. Older Cambodian physicians report treating patients for apparent primaquine-induced AHA and acute renal failure, some of whom died. Although there is no documentation of this clinical experience, the fear of primaquine toxicity persists and is the main reason why primaquine is not used in Cambodia.

Given this fear and the paucity of data on 0.75 mg/kg of weekly primaquine, we assessed the tolerability to this primaquine regimen in severely deficient, Cambodian G6PD variants to ascertain whether weekly primaquine could be given without testing for G6PDd.

## Methods

### Trial design, study site and ethics

From January 2013 to January 2014, this open parallel clinical trial in G6PDd and G6PDn vivax-infected patients was conducted at Pailin referral hospital, Pailin (Cambodian Thai border), Anlong Veng referral hospital, Anlong Venh, Oddar Meanchey (north-west Cambodia), and Pramoy health centre, Veal Veng, Pursat (west Cambodia). Ethical approvals were obtained from the National Ethical Committee for Health Research of the Cambodian Ministry of Health and the World Health Organization (WHO) Office of the Western Pacific Region. The Australia New Zealand Clinical Trials Registry number is ACTRN12613000003774.

### Participants and enrolment

The inclusion criteria were all of: (1) male or non-pregnant female aged >1 year; (2) weight ≥10 kg; (3) presentation with acute (≤10 days), symptomatic (fever or history of fever), uncomplicated, *P. vivax* infection (mono- or mixed species infection); (4) ≥2 vivax asexual parasites after reading 200 thick blood film fields; (5) written or verbal informed consent; (6) able and willing to participate; and (7) not currently taking any drugs or herbal remedies likely to cause haemolysis in G6PDd.

The exclusion criteria were any one of: (1) Hb concentration <8 g/dL; (2) malaria danger sign/s (e.g. persistent vomiting, ≥2 convulsions in previous 24 hours, prostration [23]), (3) clinically significant disease requiring treatment or further investigation; (4) on a QTc interval prolonging drug; (5) family history of cardiac related, sudden unexpected death; (6) pregnant, planning pregnancy or breast feeding; (7) for a G6PDd child <5 years – living >25 km from the research site; (8) allergic to or previous contraindicating adverse event to primaquine or dihydroartemisinin/piperaquine (DHAPP), and (9) taken an investigational drug within the previous 8 weeks.

### Conduct of clinical trial

Patients presenting to the research team who gave consent were assessed for study entry with the following: (1) brief history and examination, (2) Giemsa-stained malarial thick and thin blood films, (3) Hb concentration (HemoCue AB, Ängelholm, Sweden), (4) G6PD status using the fluorescent spot test (FST) (G-6-PDH - Spot Test, Trinity Biotech, Plc, St. Louis, USA), and (5) a urine pregnancy test to detect beta human chorionic gonadotrophin (Biotest, Selangor, Malaysia).

Enrolled patients were admitted for the first 72 hours and had: (1) a detailed history and physical examination; (2) twice daily vital sign checks; (3) blood taken for (a) full blood and reticulocyte counts (RETc) (CellDyn 3200analyser, Abbott, Rungis, France), (b) G6PD enzyme quantification (Trinity Biotech Quantitative G6PD assay) adapted on the Integra 400 analyser (Roche Diagnostic, Meylan, France) [20], (c) G6PD genotyping by an in-house polymerase chain reaction (PCR) [20], (d) Hb electrophoresis (MINICAP system, Sebia, Norcross, France) [20], (e) routine biochemistry—including haptoglobin and lactate dehydrogenase (LDH), and (f) plasma Hb (Plasma Hb Photometer, HemoCue AB, Ängelholm, Sweden); (4) blood films for malaria (D1-3, 7, 14–56), red cell morphology and manual RETc; (5) methaemoglobin estimation (Masimo oximeter, Irvine, CA, USA); and (6) urine colour graded 1–10 by the research team using a colour chart [23] (urine colour was graded every time patients passed urine as inpatients and if they were able to produce a urine sample at the follow-up visits). Blood tests a, b and e were performed on D0, 7, 28 and 56 and samples transported in a cool box to the Pasteur Institute in Phnom Penh for analysis. Vivax parasitaemia was quantified (N/μL) as the number of vivax parasites per 200 white cells on a thick blood film, assuming a total white cell count of 8,000/μL. A thick blood film was declared negative after counting 200 thick fields. Discharged patients were followed up on D7, then weekly to D56. Health workers went in search of non-attendee patients.

### G6PD enzyme activity and G6PD status

G6PD enzyme activity was classified as I to V according to the measured G6PD activity expressed as a percentage of population median [20]. G6PD status was determined by the results of the G6PD genotyping as wild type, G6PDd hemizygote male, G6PDd homozygous female or G6PDd heterozygous female. For all G6PDd patients, DNA was extracted from the buffy coat using the QIAamp DNA Blood Mini Kit (Qiagen, Courtaboeuf, France), according to the manufacturer’s instructions. DNA was used to detect the most frequent mutations in the G6PD gene by a PCR/sequencing approach [20]: (1) in exon 6 for the Mahidol (487G>A), Mediterranean (563C>T) and Coimbra (592C>T) variants; (2) in exon 9 for the Viangchan (871G>A) and Chinese-5 (1024C>T) variants; (3) in exon 11 for the Union (1360C>T) variant; and (4) in exon 12 for the Canton (1376G>T) variant [20].

### Drug treatments and allocation

We used DHAPP produced by Holley-Cotec, Beijing, under the brand name Duo-Cotecxin. Before being distributed in the health system, samples from new batches of Duo-Cotecxin were sent for analysis to an independent laboratory by the WHO Cambodia Office and found to be satisfactory. DHAPP was given once daily on D0, 1 and 2 by weight, as per the manufacturer’s instructions and 2012 National Treatment Guidelines: one tablet for 10 to <19 kg, 1.5 tablets for 19 to <30 kg, two tablets for 30 to <40 kg, three tablets for 40 to <79 kg, and four tablets for ≥80 kg. One DHAPP tablet contains 40 mg of DHA and 320 mg of PP. Primaquine (15 mg primaquine base/tablet) was given on D0 with the first dose of DHAPP and weekly thereafter for eight doses. The dosing regimen was designed by the research team (Table 1) and the target dose was 0.75 mg/kg of PQ base. Primaquine was first obtained from Cipla, India, and underwent satisfactory quality control at an external laboratory. Towards the end of the study, primaquine was obtained from the Government Pharmaceutical Organisation, Thailand, but not sent for external quality control.

**Table 1:** Dose of weekly primaquine expressed as milligrams of primaquine base

Weight (kg)	Number of primaquine tablets	Primaquine dose (mg)	Primaquine dose (mg/kg)
10–17	0.5	7.5	0.44–0.75
18–25	1	15	0.6–0.83
26–35	1.5	22.5	0.64–0.87
36–45	2	30	0.67–0.83
46–55	2.5	37.5	0.69–0.82
56–75	3	45	0.64–0.82
≥76	4	60	≤0.78

If vomiting occurred within 30 minutes, a full dose of either or both drugs was re-administered; if between 31 and 60 minutes, half doses were given. All treatments were given supervised by study nurses. Other drugs were allowed as clinically indicated, e.g. paracetamol for fever.

### Rescue treatment

Patients who failed treatment (i.e. had a persistent or a recurrent vivax parasitaemia) were retreated with oral DHAPP. Those who developed falciparum malaria during follow-up were treated with atovaquone/proguanil, as per National Guidelines. Patients with persistent vomiting or severe malaria [24] received intravenous artesunate or, if unavailable, intramuscular artemether followed by oral treatment as above.

### Outcomes

The primary outcome was patients completing all eight primaquine doses, i.e. not having primaquine stopped because of primaquine toxicity, defined by research team consensus as any one of: (1) a >25 % fall in baseline Hb by D7, (2) severe anaemia by D7 (Hb <7 g/dL for all ages), (3) haemoglobinuria (urine colour ≥8, using a urine colour chart graded 1 to 10 [23]) for 2 days, (4) methaemoglobinaemia >20 %, (5) a >50 % increase in creatinine from D0 with evidence of AHA, and (5) AHA necessitating a blood transfusion (added *post hoc*).

Secondary endpoints included: (1) changes over time of whole blood and plasma Hb concentrations and routine biochemical parameters, and (2) incidence of adverse events (AEs).

### Adverse events and safety monitoring

AEs and serious AEs (SAEs) were defined and graded according to the 2004 US National Institutes of Health Division of AIDS toxicity table [25]. All SAEs were to be reported within 24 hours to the study principal investigator and ethics committees. An independent Drug Safety and Monitoring Board (DSMB) monitored study safety; data from G6PDd patients was sent to the DSMB as they became available.

### Sample size

Sample size was based on demonstrating a difference in primaquine-related toxicity between the two G6PD groups. Such data are lacking in vivax-infected patients, so, assuming similar Hb dynamics between *P. vivax* and *P. falciparum* [26], we used a falciparum database of South-East Asian patients treated with artemisinin-based combinations; 374/6,882 (5.4 %) had a fall in baseline haematocrit ≥25 % by D7. We hypothesised primaquine toxicity rates of 5 % (G6PDn) and 25 % (G6PDd). Using a power of 0.8, a two-sided alpha of 0.05, and a 2:1 allocation in favour of the G6PDd arm, the sample size was 92 and 46 patients, rounded up to 100 G6PDd and 50 G6PDn patients. Between ~580 and ~1,200 patients would need to be screened to recruit 100 G6PDd patients, based on reported G6PDd prevalence rates [19].

### Data management and statistical methods

Data were entered onto standardised case record forms, checked against source documents, double entered into Epidata, and analysed using Stata v13 (Stata Corporation, College Station,mTX, USA). Proportional data were compared using chi-squared or Fisher’s exact tests, as appropriate, and continuous data by student’s *t* (normally distributed data) or Mann–Whitney U (skewed data) tests. The relationship between the fractional fall in Hb on D7 versus baseline and the mg/kg dose of primaquine was assessed by Spearman rho test (skewed data), and with the baseline G6PD enzyme activity by Pearson’s correlation coefficient (transforming the G6PD data to become normally distributed).

## Results

### Patient disposition and baseline characteristics

**Fig. 1:**
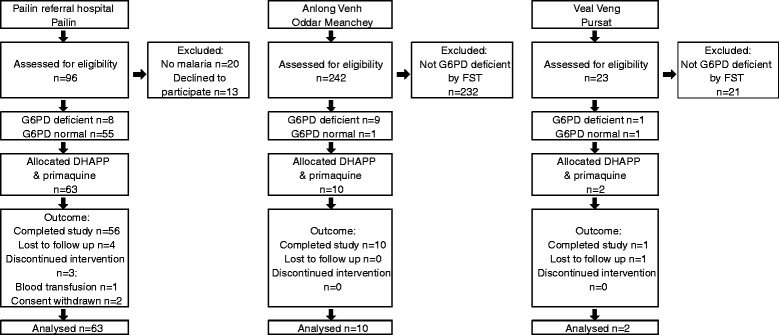
Trial profile. G6PD status was determined initially using the fluorescent spot test (*FST*). At Anlong Venh and Veal Veng, only FST-diagnosed G6PDd patients were recruited. Final G6PD status shown here is based on G6PD enzyme activity and G6PD genotype. *DHAPP* dihydroartemisinin/piperaquine, *G6PD* glucose-6-phosphate dehydrogenase

From January 2013 to January 2014, 361 patients were screened and 75 with mono vivax infections were enrolled into the study; eight did not complete follow-up (Fig. 1). Because the sample size requirement for G6PDn was met in Pailin, we only recruited patients at the other two sites if the FST result showed they were G6PDd. Most patients were male (n = 63) of median age 24 years [range 5–63, interquartile range (IQR) 9–46]. Median female age was 29 years (range 9–56, IQR 15–45). Fifteen patients (20 %) were <18 years of age (Table 2). A total of 18 patients were G6PDd: 17 had the Viangchan variant (14 hemizygous males, 3 heterozygous females), and one male had the Canton variant.

**Table 2:** Baseline characteristics in the two glucose-6-phosphate dehydrogenase groups

Parameter	G6PD deficient	G6PD normal	*P* value
	n = 18	n = 57	
Age (years)	25 (5–56)	24 (7–63)	0.95
Aged <18 years	5 (27.8)	10 (17.5)	0.34
Male, female	15 (83.3), 3	48 (84.2), 9	0.93
Weight (kg)	54 (20–56)	53 (14–88)	0.83
Symptoms			
Headache	14 (77.8)	53 (92.9)	0.07
Fever	14 (77.8)	48 (84.2)	0.53
Chills	12 (66.7)	47 (82.5)	0.15
Abdominal pain	4 (22.2)	28 (49.1)	0.04
Nausea	3 (16.7)	14 (24.6)	0.48
Cough	1 (5.6)	7 (12.3)	0.42
Abnormal urine colour	9 (50)	7 (12.3)	0.001
Signs			
Mean (SD) temperature (°C)	37.8 (0.9)	38.6 (1.0)	0.002
Mean (SD) pulse (mmHg)	92 (18.6)	96 (11.1)	0.32
Mean (SD) systolic BP (mmHg)	105 (15.5)	110 (12.4)	`0.17
Mean (SD) diastolic BP (mmHg)	61 (11)	66 (10)	0.09
Normal conjunctival colour	18 (100)	56 (98.3)	0.57
Pale palms	1 (5.6)	5 (8.8)	0.66
Palpable liver	1 (5.6)	2 (3.5)	0.69
Palpable spleen	1 (5.6)	2 (3.5)	0.69
Haematological parameters			
G6PD activity (U/g Hb)	0.85 (0.1–1.5)^a^	12 (6.9–18.5)	0.000
Normal haemoglobin	11 (61.1)	31 (54.4)	
Heterozygous HbE	5 (27.8)	20 (35.1)	
Homozygous HbE	0	1 (1.75)	
Alpha/beta thalassaemia	1 (5.6)	5 (8.7)	0.4
Haemoglobin (g/dL)	13.0 (9.6–16)	13.5 (9–16.3)	0.46
Total white cell count ×10^3^/μL	5.15 (1.4–8.3)	5.0 (1.1–11.5)	0.61
Platelet count ×10^3^/μL	126 (42–187)	99 (12–204)	0.26
Vivax parasitaemia/μL	6,420 (159–9326)	7,888 (220–59,542)	0.13

Unless otherwise stated, continuous data are shown as median (range)

Proportional data are shown as N (%)

*BP* blood pressure, *G6PD* glucose-6-phosphate dehydrogenase *HbE* haemoglobin E, *SD* standard deviation

^a^substituting the two missing enzyme values with post D0 values: 0.9 (0.1–2.6) U/g Hb

**Fig. 2:**
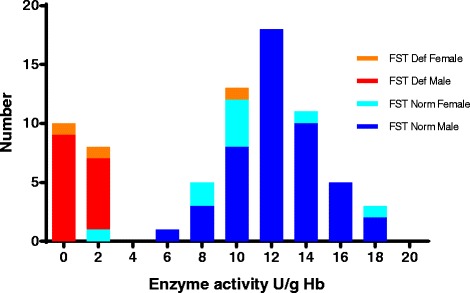
Results of the fluorescent spot test as a function of the measured G6PD enzyme activity. One G6PD enzyme value was unavailable for a fluorescent spot test (*FST*)-diagnosed G6PD deficient male who was confirmed G6PD wild type by polymerase chain reaction

Baseline demographic, clinical and laboratory characteristics were similar between the two G6PD groups (Table 2), except for reported rates of abdominal pain, abnormal urine colour, mean body temperature and G6PD activity. Four PCR-determined G6PD wild-type patients had low G6PD enzyme activities that were probably due to delayed measurement; in three patients, the baseline values were inconsistent with later G6PD activity values and in one there were no other G6PD activity values. All such values have been excluded from Table 2. Two G6PDd patients had missing baseline enzyme activity values (also excluded from Table 2) but were classified using post D0 G6PD enzyme activity results. Of the 18 G6PDd patients, 13 were class II (1 to <10 % population median of 12 U/g Hb) and five were class III (≥10 to 60 %) G6PDd. Three patients had discordant FST results: two from Anlong Venh and Veal Veng were diagnosed as FST G6PDd but were subsequently confirmed as G6PD wild type and one FST-diagnosed G6PD normal patient was later confirmed PCR G6PDd (Fig. 2).

### Primary outcome

One 23-year-old G6PDd male, diagnosed subsequently with G6PDd Viangchan, had a SAE—clinically significant anaemia that was probably primaquine related and was treated by blood transfusion. His D0 Hb fell from 10.0 to 7.5 g/dL on D3 and to 7.2 g/dL on D5, for a fractional fall of 28 %. He developed slowly progressive dyspnoea and by D5 he was breathless walking the short distance from his bed to the toilet. He did not have gross haemoglobinuria—his darkest urine colour was graded 4. He was not given further primaquine. When questioned later, he said he went to a village shop for stomach ache and fever and was advised by the drug seller to take cimetidine and ciprofloxacin; he took two doses of both cimetidine 400 mg and ciprofloxacin 500 mg one day before enrolment but had not reported this to the study team at enrolment.

On D3, 4/18 (22.2 %) G6PDd versus 0/57 G6PDn had a >25 % fall in Hb (*P* = 0.003); all four patients were G6PDd Viangchan: three hemizygous males and one heterozygous female. Their D0–D3 Hb concentrations were: 16.0–11.3, 14.8–9.0, 12.8–9.1 and 13.2–9.8 g/dL. Compared to the G6PDn group, the median (range) fractional fall in haematocrit was significantly higher (*P* = 0.0001) in the G6PDd group: −15.26 % (−39.1 to 3.6) versus −6.15 % (−19.1 to 22.8).

On D7, two of the males with >25 % fall in Hb on D3 had persistent fractional falls in haemoglobin >25 %: 14.8–9.9 and 12.8–8.9 g/dL. Compared to the G6PDn group, the median fractional fall in Hb was significantly higher (*P* = 0.0002) in the G6PDd group: −16.3 % (−33.1 to 6.5) versus −3.7 % (−17.5 to 24.3). Primaquine was not stopped in these two male patients because they were well.

By D7, the cumulative number of patients with protocol-defined primaquine toxicity (i.e. including those identified on D3 and the transfused male) was 5/18 G6PDd (27.7 %) versus 0/57 from the G6PDn group (*P* = 0.00049). This difference between the two arms was still significant if the two D3 patients with transient PQ toxicity were excluded: 3/18 (16.6 %) versus 0 % (*P* = 0.01). No patients developed severe anaemia, haemoglobinuria, methaemoglobin >4.9 % or AHA-related acute kidney injury.

### Secondary outcomes

**Fig. 3:**
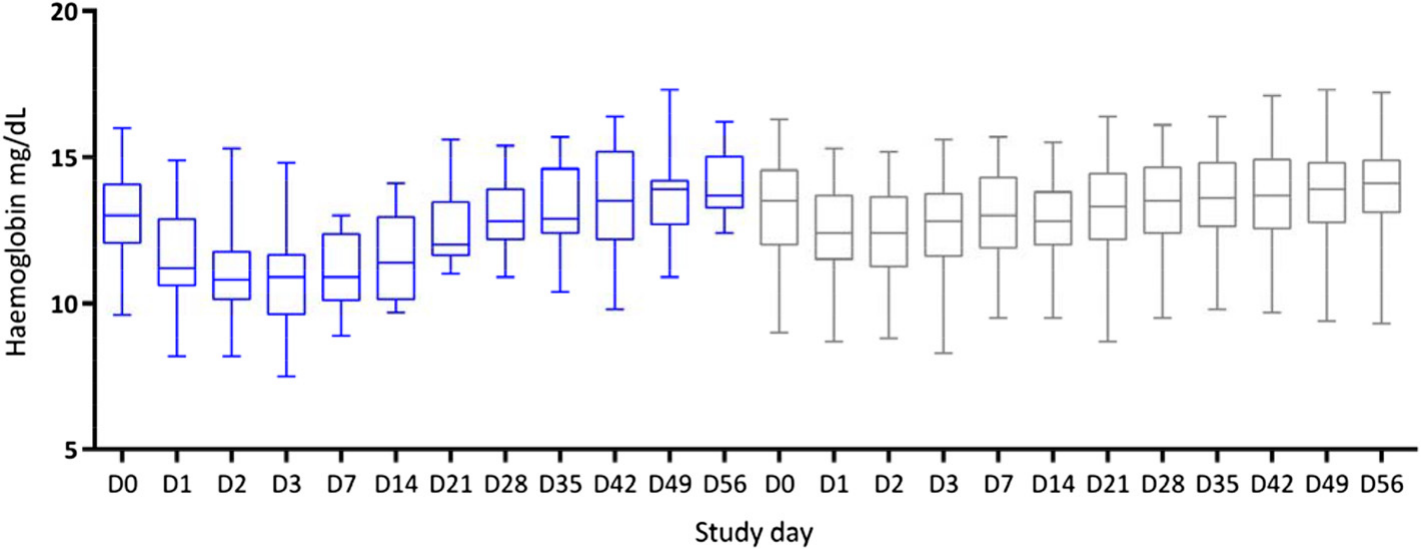
Boxplots (median, interquartile range, full range) of HemoCue-measured haemoglobin (*Hb*) concentrations over time as a function of G6PD status. Post transfusion Hb concentrations have been excluded. *Blue* box plots are G6PD-deficient patients. *G6PD* glucose-6-phosphate dehydrogenase

The median nadir Hb concentration occurred on D2 in both groups and began to rise on D3 in the G6PDn group and D14 in the G6PDd group (Fig. 3, Table 3). The days of the greatest decline in the absolute median Hb concentration and fractional median change in Hb concentrations were D2 and D7, respectively (Fig. 3, Additional file 1). The differences in these two parameters by G6PD status were statistically significant for the first 14 days of follow-up (Table 3). The largest median (range) Hb difference (*P* = 0.0002) was on D7: −2.2 g/dL (−4.9 to 0.8, G6PDd) versus −0.5 g/dL (−2.2 to 2.8, G6PDn), ∆ = −1.7 g/dL (−2.7 to 2.0). The fractional fall in Hb on D7 versus baseline was unrelated to the mg/kg dose of administered primaquine in the G6PDn (*P* = 0.68) or the G6PDd group (*P* = 0.77) but was associated weakly with baseline G6PD enzyme activity (*P* = 0.013), for a coefficient of variation of ~8 %. Hb recovery to the median D0 Hb occurred on D28 (G6PDn) and D35 (G6PDd, Fig. 2); the median recovery time for individual patients was 28 days for both arms (*P* = 0.48). Out of the 63 patients, 14 (22.2 %) had lower median Hb concentrations on D56 versus D0, which was unrelated to G6PD status (*P* = 1.0).

**Table 3:** Changes in haemoglobin concentrations in during the first 28 days of follow-up

Day	G6PD deficient	G6PD normal	*P*	G6PD deficient	G6PD normal	*P*	G6PD deficient	G6PD normal	*P*
	Haemoglobin concentration (g/dL)			Changes in haemoglobin concentrations versus day 0			Fractional changes in haemoglobin concentrations versus day 0		
0	13.0 (9.6 to 16)	13.5 (9 to 16.3)	0.46	–	–		–	–	
1	11.2 (8.2 to 14.9)	12.4 (8.7 to 15.3)	0.041	– 1.5 (–4.0 to 1.1)	– 0.8 (– 3.2 to 3.3)	0.059	–12.5 (– 27.1 to 8.7)	– 6.5 (– 22.7 to 28.7)	0.028
2	10.8 (8.2 to 15.3)	12.4 (8.8 to 15.2)	0.006	– 1.7 (–5.0 to 0.6)	– 1 (– 3.5 to 2.1)	0.047	–13.3 (– 31.2 to 4.1)	– 7.4 (– 23.6 to 25.2)	0.029
3	10.9 (7.5 to 14.8)	12.8 (8.3 to 15.6)	0.0002	– 1.9 (–5.8 to 0.4)	– 0.8 (– 2.6 to 2.9)	0.0003	–15.3 (– 39.2 to 3.6)	– 6.1 (– 19.1 to 22.8)	0.0001
7	10.9 (8.9 to 13)	13 (9.5 to 15.7)	0.0001	– 2.2 (–4.9 to 0.8)	–0.5 (– 2.2 to 2.8)	0.0002	– 16.3 (– 33.1 to 6.5)	– 3.7 (– 17.5 to 23.3)	0.0002
14	11.4 (9.7 to 14.1)	12.8 (9.5 to 15.5)	0.0067	– 1.7 (–3.0 to 0.3)	–0.5 (– 4.2 to 2.1)	0.005	– 12.9 (– 22.7 to 2.7)	– 4 (– 25.7 to 18.1)	0.0035
21	12 (11 to 15.6)	13.3 (8.7 to 16.4)	0.039	– 0.6 (–2 to 1.6)	0.3 (– 3.5 to 2.1)	0.1	– 4.7 (– 15.1 to 14.6)	2.1 (– 24.3 to 16.8)	0.11
28	12.8 (10.9 to 15.4)	13.5 (9.5 to 16.1)	0.26	0.2 (–2 to 2.6)	0.3 (–2.3 to 2.7)	0.62	1.5 (– 13.6 to 27.1)	2.3 (– 15.8 to 21.2)	0.58

All data are shown as median (range)

*G6PD* glucose-6-phosphate dehydrogenase

G6PDd patients had higher median LDH concentrations and proportions with values above the upper limit of normal, higher median RETc on D7, and lower median haptoglobin concentrations on D7–D56 (Table 4). Plasma Hb, serum unconjugated bilirubin and creatinine concentrations were similar between both groups. All patients cleared their vivax parasites by D2 and none had recurrent malaria during follow-up.

**Table 4:** Changes in laboratory parameters over time

Parameter	G6PDd	G6PDn	*P*	G6PDd	G6PDn	*P*	G6PDd	G6PDn	*P*	G6PDd	G6PDn	*P*
	D0			D7			D28			D56		
Reticulocyte count (%)	1.7	1.3	0.051	3	1.9	0.0009	1.4	1.5	0.056	1	1.2	0.98
	(0.6–3.8)	(0.5–4.5)		(1.6–3)	(0.6–7)		(0.9–4.8)	(0.3–6)		(0.5–2.4)	(0.4–2.6)	
Plasma haemoglobin (g/dL)	0.1	0.085	0.3	0.05	0.05	0.81	0.03	0.04	0.75	0.08	0.08	0.9
	(0.01–13.5)	(0.01–0.71)		(0.02–0.46)	(0–0.27)		(0.01–0.13)	(0–0.28)		(0.04–0.09)	(0–0.24)	
Haptoglobin (g/L)	0.34	0.59	0.21	0.35	0.72	0.006	0.69	0.98	0.03	0.54	0.87	0.029
	(0–1.48)	(0–2.16)		(0.09–1.07)	(0.1–1.83)		(0.16–1.19)	(0.04–2.32)		(0.08–1.25)	(0.06–2.22)	
Low haptoglobin^a^	8/16	14/56	0.056	5/14	8/57	0.11	2/14	3/52	0.28	5/14	4/49	0.02
	(50)	(25)		(35.7)	(14.1)		(14.3)	(5.8)		(35.7)	(8.2)	
LDH (IU/L)	308	231	0.01	350	196	0.0002	319.5	183.5	0.005	296.5	181	0.008
	(127–800)	(23–611)		(178–700)	(58–783)		(121–619)	(37–593)		(154–746)	(118–522)	
High LDH^a^	13/17	18/56	0.002	12/15	12/57	0.000	10/14	7/52	0.001	8/14	7/49	0.002
	(76.5)	(32.1)		(80)	(21.1)		(71.4)	(13.5)		(57.1)	(14.3)	
Unconjugated bilirubin (mg/L)	2.5	3.75	0.63	1.7	1.7	0.9	1.4	1.4	0.66	1.45	1.5	0.59
	(0.6–9.8)	(0.5–14.3)		(0.4–4.8)	(0.19–3.1)		(0.7–3.6)	(0.2–4.3)		(0.6–3.4)	(0.5–4.3)	
Creatinine (μmol/L)	58	74	0.003	58	63	0.13	54.5	60.5	0.29	54.5	58	0.32
	(28–86)	(35–90)		(28–83)	(28–85)		(19–82)	(27–99)		(24–80)	(26–84)	

^a^numerator/denominator (%)

*Low* below the lower limit of normal for haptoglobin LLN (0.3 g/L), *High* above the upper limit of normal for LDH (255 IU/L)

*G6PDd* glucose-6-phosphate dehydrogenase deficient, *G6PDn* glucose-6-phosphate dehydrogenase normal, *LDH* lactate dehydrogenase

### Harms and adverse events

Patients tolerated their treatments well. Nineteen patients had a total of 38 clinical AEs; 19 (50 %) of these AEs occurred on D0–2 (Table 5). Most were mild and considered unrelated or unlikely to be primaquine related. One G6PDn patient had early vomiting on D0 and was re-dosed without further incident.

**Table 5:** Summary of reported or detected clinical adverse events and their relationships to study drugs

Adverse event	Unrelated or unlikely related			Possibly or probably related		
	Severity			Severity		
	Mild	Moderate	Severe	Mild	Moderate	Severe
Abdominal pain	4	1				
Epigastric pain		2		1		
Anorexia				1		
Nausea	1			1		
Vomiting				1	1	
Diarrhoea	2					
Jaundice				1		
Pale conjunctivae					2	
Anaemia						1
Back pain		1				
Change in urine colour					1	
Headache	4	1				
Dizziness				2		
Hearing loss	1			1		
Asthenia	2			1	2	
Sore throat	1					
Fever		1				
Chills		1				

## Discussion

This is the first study to evaluate the tolerability of a weekly anti-relapse primaquine regimen in patients with acute vivax malaria and South-East Asian variants of G6PDd. Within the first week, approximately one quarter of G6PDd patients experienced substantial drops (>25 %) in their Hb concentrations, including one patient whose progressive fall in Hb necessitated a blood transfusion. These results preclude the use of unsupervised weekly primaquine in settings where severe G6PDd is present, and mandate prior testing for G6PDd.

The male patient who required a blood transfusion had not mentioned his visit to the village shop where he was advised to take cimetidine and ciprofloxacin. Cimetidine is a known cytochrome P450 3A4 inhibitor [27] and ciprofloxacin has been implicated in one case report of AHA in a G6PDd patient [28]. Thus, a drug–drug interaction and/or direct red cell toxicity may have contributed to his worsening anaemia. This is a reminder for clinicians to be vigilant when prescribing primaquine to patients already taking drugs that could enhance the haemolytic potential of primaquine.

Our study focused on the most vulnerable group of patients at risk of AHA. The criteria of primaquine toxicity were selected to detect events that could potentially be dangerous to unsupervised patients who make up the vast majority in malaria-endemic countries. All of our G6PDd hemizygous males and the three heterozygous females experienced a fall in Hb during follow-up, especially within the first week. However, aside from the transfused G6PDd male, none had significant symptoms of anaemia and all had later increases in their Hb concentrations despite continued dosing. Indeed, primaquine was continued in the two G6PDd males with fractional Hb falls >25 % on D7 because they were clinically well and had adequate Hb concentrations of ~9 g/dL. As expected, the G6PDn patients tended to have smaller declines in Hb concentrations and they tolerated weekly primaquine despite some of them experiencing drops in Hb exceeding 2 g/dL.

The key to the safe use of primaquine and, in the future, tafenoquine as anti-relapse treatment to achieve *P. vivax* radical cure is the accurate diagnosis of G6PDd and identifying those with more severe G6PDd. Indeed, tafenoquine registration trials are excluding patients with enzyme activities <70 % of the population median (NCT02216123) [29]. In our setting, a patient with severe G6PDd who could be misclassified as G6PDn and would thus receive the appropriate primaquine anti-relapse dose of 0.5 mg/kg/d (30 mg in an adult) would probably develop severe AHA [12, 17, 22, 30]. G6PDd testing is currently laboratory-based in Cambodia but the wider availability of a promising and robust point-of-care rapid diagnostic test (RDT) [31] capable of detecting patients with G6PD enzyme activities <30 % (<3.6 U/gHb) of the Cambodian median (i.e. those at the lower end of the G6PD activity spectrum) would open up the option of G6PDd testing by village malaria workers (VMWs), referring RDT-diagnosed G6PDd patients for medical supervision and treating the other patients in the community. Such a strategy should be piloted to assess its feasibility, VMW acceptability, cost, efficacy and safety.

In 2012, the WHO recommended the use of single low-dose primaquine (0.25 mg/kg) without testing for G6PDd to block the transmission of artemisinin-resistant *P. falciparum* [32]. Limited evidence at that time suggested this dose would be tolerated in all G6PDd patients. Our findings support this notion and suggest that 0.25 mg/kg would be tolerated well in falciparum-infected Cambodians with severe G6PDd without the need for testing for G6PDd. This should help inform a decision to deploy low-dose primaquine, especially in Cambodia, the epicentre of artemisinin-resistant *P. falciparum* [15, 33].

This study had limitations. The total number of G6PDd patients was only 18, most were hemizygous males, and most had the Viangchan variant; their measured enzyme activities were low (median <1 U/g Hb), placing them at the severe end of the G6PD spectrum. Perhaps surprisingly, this study is currently the largest clinical series of primaquine-treated, vivax-infected patients with mostly severe G6PDd. Our findings are consistent with other smaller primaquine challenge studies (0.75 mg/kg) in otherwise healthy volunteers with Mediterranean G6PDd and patients who had Hb falls of approximately 20–25 % [12, 34–37]. More safety data are needed from larger safety studies in patients with different G6PDd variants and in heterozygous G6PDd females who represent a therapeutic challenge. The small number of G6PDd patients recruited meant that the original G6PDd sample size (100) was far from achieved. Limited data suggest that Mediterranean variant G6PDd protects against clinical *P. vivax* disease [38, 39] so researchers need to take this possibility into account when planning studies. Despite the loss of power, key analyses still yielded significant comparisons. Adult males made up the majority of recruited patients, in keeping with the malaria epidemiology in Cambodia, and patients with a baseline Hb of <8 g/dL were excluded. Therefore, the findings of this study must be applied with caution to children and cannot be extrapolated to those with lesser degrees of G6PDd or to adults and children with moderately severe anaemia of <8 g/dL. The latter is an urgent group for further research.

## Conclusions

This is the first study to evaluate weekly primaquine in vivax-infected patients with low or very low G6PD enzyme activities. In our setting, primaquine should not be given as anti-relapse treatment without knowing the G6PD status of patients and should be given under medical supervision to those found to be G6PDd. Other National Malaria Control Programmes should assess weekly primaquine in G6PDd patients to inform their elimination strategies.

## Additional file

Additional file 1:
**Fractional changes in haemoglobin concentrations on given follow-up days versus baseline over time as a function of G6PD status.** Post transfusion haemoglobin concentrations have been excluded. Blue box plots (left) are glucose-6-phosphate deficient patients. D10 = Hb on D1 − Hb on D0 × 100 % / Hb on D0. (DOCX 345 kb)

## Abbreviations

AE: adverse event
AHA: acute haemolytic anaemia
AIDS: acquired immune deficiency syndrome
D: day
DHAPP: dihydroartemisinin/piperaquine
DSMB: Drug Safety & Monitoring Board
FST: fluorescent spot test
G6PD: glucose-6-phosphate dehydrogenase
G6PDd: glucose-6-phosphate dehydrogenase deficiency/deficient
G6PDn: glucose-6-phosphate dehydrogenase normal
Hb: haemoglobin
LDH: lactate dehydrogenase
PCR: polymerase chain reaction
RDT: rapid diagnostic test
RETc: reticulocyte count
SAE: serious adverse event
VMW: village malaria workers
