# Supplementary figures and images for: Tolerability and safety of weekly primaquine against relapse of Plasmodium vivax in Cambodians with glucose-6-phosphate dehydrogenase deficiency
Source: BMC Med. 2015 Aug 25;13:203. doi: 10.1186/s12916-015-0441-1 (PMC4549079; doi:10.1186/s12916-015-0441-1)

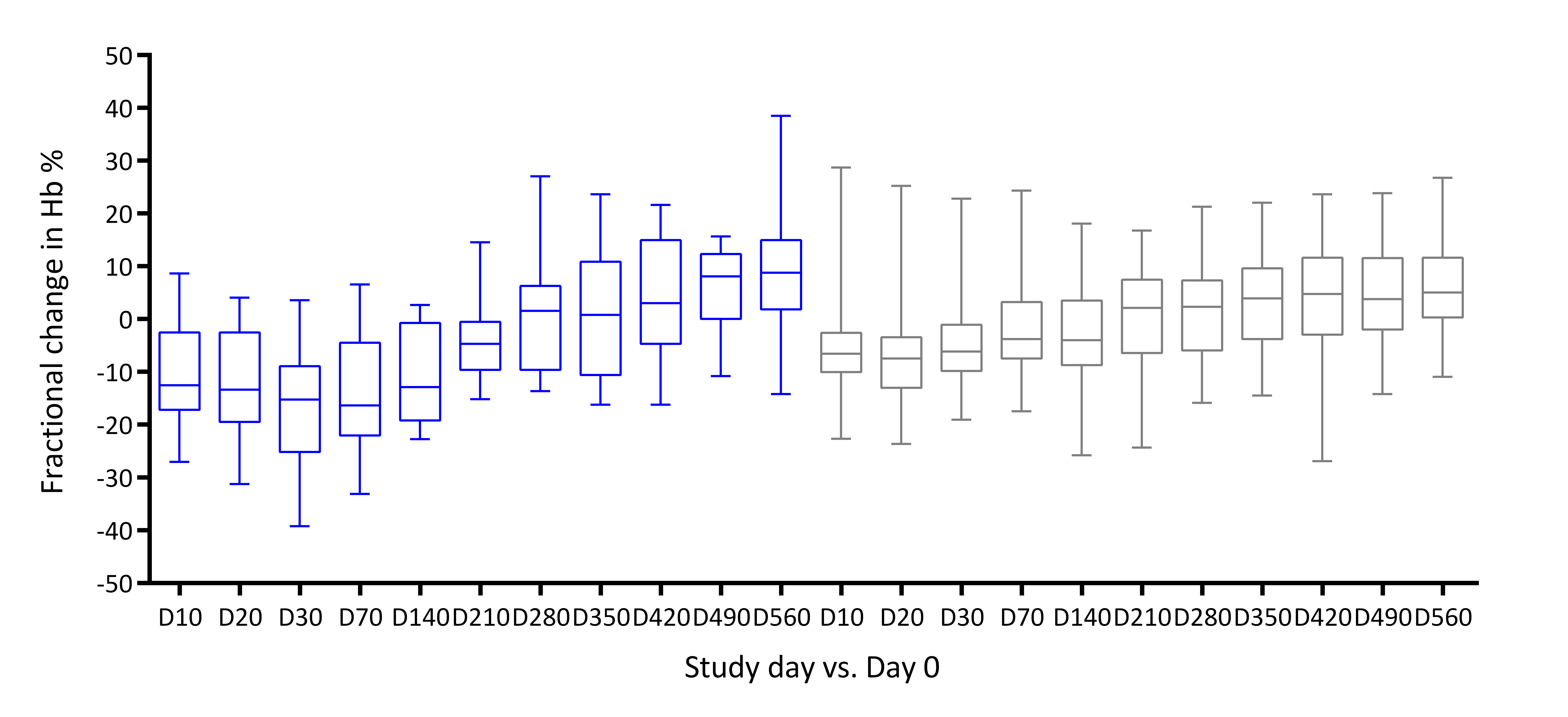

Supplement: Additional file 1: — Fractional changes in haemoglobin concentrations on given follow-up days versus baseline over time as a function of G6PD status. Post transfusion haemoglobin concentrations have been excluded. Blue box plots (left) are glucose-6-phosphate deficient patients. D10 = Hb on D1 − Hb on D0 × 100 % / Hb on D0. (DOCX 345 kb) [file 12916_2015_441_MOESM1_ESM.docx]
